# Can Nicotinamide Adenine Dinucleotide (NAD^+^) and Sirtuins Be Harnessed to Improve Mare Fertility?

**DOI:** 10.3390/ani14020193

**Published:** 2024-01-07

**Authors:** Charley-Lea Pollard

**Affiliations:** Sydney School of Veterinary Science, Faculty of Science, The University of Sydney, Camden, NSW 2570, Australia; charley.pollard@sydney.edu.au

**Keywords:** fertility, mare, NAD^+^, oocyte quality, Sirtuins

## Abstract

**Simple Summary:**

The reproductive capacity of mares has suffered drastically as a result of years of selection based on athletic performance. The world of equine reproduction is now trying to catch up. Recent studies examining niacin deficiencies in women have indicated that nicotinamide adenine dinucleotide (NAD^+^) is vital for embryo and foetal development, with further animal models showing improvements to oocyte quality with the treatment of NAD^+^ precursors. Given the enormous benefits shown in these studies, these results show great promise in improving reproductive outcomes in mares.

**Abstract:**

Years of sire and dam selection based on their pedigree and athletic performance has resulted in a reduction in the reproductive capability of horses. Mare age is considered a major barrier to equine reproduction largely due to an increase in the age at which mares are typically bred following the end of their racing career. Nicotinamide adenine dinucleotide (NAD^+^) and its involvement in the activation of Sirtuins in fertility are an emerging field of study, with the role of NAD^+^ in oocyte maturation and embryo development becoming increasingly apparent. While assisted reproductive technologies in equine breeding programs are in their infancy compared to other livestock species such as cattle, there is much more to be learnt, from oocyte maturation to early embryo development and beyond in the mare, which are difficult to study given the complexities associated with mare fertility research. This review examines what is already known about the role of NAD^+^ and Sirtuins in fertility and discusses how NAD^+^-elevating agents may be used to activate Sirtuin proteins to improve equine breeding and embryo production programs both in vivo and in vitro.

## 1. Introduction

The equine world of reproduction is far behind that of other industries such as the cattle, sheep and pig reproductive industries for a number of reasons. Firstly, horses are typically selected for their athletic prowess, resulting in a progressive loss of reproductive ability over the years [1,2,3]. Consequently, the production of foals in Australia has been in steady decline since 2010 [4]. A single service by a Thoroughbred stallion in the Thoroughbred industry carries a heavy cost in that a financial profit is made only when a foal has been produced in six out of seven years of a Thoroughbred mare’s reproductive life [5]. Secondly, while cattle, sheep and pigs are produced to be marketed as food after achieving the desired marketable traits or kept for breeding, horses typically begin breeding at the end of their racing career. As such, some broodmares are much older, yet their genetics can prove extremely valuable and highly sought after within the industry. Older mares, like women, exhibit a reduction in reproductive efficiency compared with their younger counterparts, representing a second devastating hit to their already reduced fertility. Thirdly, the incidence of early embryonic death (EED) is problematic (previously reported as high as 20–30%) [1,5], while its aetiology remains largely unknown and is challenging to study. Defective embryos, environmental factors and inadequate maternal factors are proposed to be the greatest contributors to embryonic losses in the mare [3,6], but the lack of available material [7] in combination with the requirement that all Thoroughbreds to be registered in the studbook must be conceived naturally makes early embryonic death difficult to investigate.

The effects of maternal nutrition and its role in oocyte quality and embryonic losses are becoming increasingly apparent [6,8] and may provide a non-invasive avenue for manipulating mare fertility. An increased incidence of congenital defects and spontaneous miscarriage in niacin-deficient human and mice subjects was attributed to a deficiency in nicotinamide adenine dinucleotide (NAD^+^) [9]. Supplementing maternal diets with NAD^+^ precursors improved oocyte quality and embryo development in aged mice [10,11], indicative of the reduction in reproductive capacity in older subjects. Since elevating the levels of NAD^+^ in oocytes and embryos both in vitro and in vivo has beneficial effects in other species [10,12,13,14,15,16,17,18,19,20,21], can reproductive efficiency in the mare be improved through promoting NAD^+^ biosynthesis via dietary interventions? There has been no evidence of niacin deficiencies in horses [22] and the additional supplementation of niacin had no effect on exercising Thoroughbred geldings [23]. However, niacin is one of the more commonly added vitamins in pelleted horse feed. There are no current recommendations for niacin requirements in equine diets [24] which are presumed to be met through production by hindgut microbiota [25,26]. On the other hand, feeding nicotinic acid at a supraphysiological dose to mares showed an increase in the concentration of various NAD^+^ metabolites over time, with some remaining elevated in the blood at the end of the trial period [27]. Additionally, NAD^+^ metabolites were also detected in elevated concentrations in the follicular fluid of mares fed nicotinic acid during the oestrous cycle [28]. While there is conflicting evidence regarding the effects of niacin supplementation in mares, the demand for niacin throughout pregnancy is much greater in humans [29], so can additional dietary niacin also prove beneficial to reproductive function in mares?

This review discusses the production of NAD^+^ in cells, its role in the activation of Sirtuins (SIRTs) and the effects on fertility, with a heavy focus on improving fertility in the mare. Recently, SIRTs were shown to improve reproductive function in mice, cattle and pigs [10,11]; however, SIRTs and NAD^+^ are yet to be studied in the horse. The high incidence of EED [1,5] in the mare is a phenomenon of major interest to the equine breeding industry as a result of the high economic cost associated with repeated services following a failed conception and a reduction in the number of foals produced per service. The cause of EED in mares is multifactorial and poorly understood, but poor oocyte quality is thought to play a major role [3,6]. Given the paucity of studies regarding the role of NAD^+^ and SIRTs in equine fertility, the potential links between NAD^+^, SIRT activation and oocyte quality in the mare are explored, linking current knowledge on the role of SIRTs and NAD^+^ in reproduction to reproductive issues in the mare.

## 2. NAD, SIRTs and Oocytes

NAD^+^ is synthesised through the absorption and conversion of tryptophan and through the metabolism of dietary niacin [30,31,32]. Tryptophan is an essential amino acid in that it must be acquired through the diet. Although most of the tryptophan absorbed from the diet is used in the synthesis of NAD^+^, the amount of tryptophan needed to produce the equivalent amount of NAD^+^ is in excess of 60 times the concentration of niacin required [33]. As such, the Preiss–Handler and salvage pathways of NAD^+^ biosynthesis are more commonly utilised for the production of NAD^+^ due to the insufficiency of tryptophan alone to maintain adequate cellular pools of NAD^+^ in mammals [34]. NAD^+^ is a cofactor in many biological processes within the cell and functions as a substrate for Sirtuin proteins. Sirtuins are a family of NAD^+^-dependent deacetylases (also referred to as class III histone deacetylases; HDACs), which consume NAD^+^ to exert their effects [35].

The role of NAD^+^ and Sirtuins in reproductive function is a relatively new area of research and is scarcely understood. There are seven mammalian Sirtuin proteins (SIRT1-7) each with various sub-cellular locations: SIRT-1, -6 and -7 are localised to the nucleus [36]; SIRT2 is localised to the cytoplasm [37,38]; and SIRT3-5 are localised to the mitochondria [36]. While there appears to be some redundancy in the effects between each of the proteins, it is evident that Sirtuins play important roles in meiosis and metabolic function across a range of species as reviewed by Pollard et al. and Tatone et al. [32,39], although precise mechanisms remain to be elucidated. In contrast, the effects of NAD^+^-elevating treatments appear species-specific, with spatial and temporal effects observed within species. Studies of the role of NAD^+^ in reproductive function primarily focussed on the addition of NAD^+^ precursors to oocytes during oocyte maturation in in vitro cattle, mice and pigs [10,11,12,13,14,15,16,17,18,40,41,42,43,44,45], while very few utilised NAD^+^ precursors in mice, cattle, humans and pigs in vivo [9,10,11,18,19], and no studies have demonstrated a direct link to Sirtuin mechanistic function. To better understand the role of NAD^+^ in Sirtuin function in oocytes and embryos, studies have previously relied on specific knock out models to elucidate their effects, opting to disrupt either NAD^+^ biosynthesis or knock out specific Sirtuin proteins.

RNA sequencing analysis has shown that all seven Sirtuins are expressed in the oviduct of the mare with a downregulation in the expression of *Sirt6* in the ipsilateral ovary of pregnant mares compared with normal cycling mares [46]. Combined with a lack of *Sirt6* expression in the trophectoderm and inner-cell mass of equine embryos [47], these data indicate that SIRT6 plays a more important role during oocyte maturation and fertilization than in embryo development. Additionally, *Sirt1* and *Sirt5* expression was upregulated in the inner-cell mass and trophectoderm, with no differences in *Sirt2* expression [47], indicative of the importance of SIRT-1 and -5 in the formation of the embryo proper. Interestingly, *Sirt-1*, *-2*, *-3*, *-5* and *-7* expression decreased in the endometrium from mid- to late gestation in the mare, with *Sirt-1*, *-2* and *-6* expression in the chorioallantois and *Sirt-1-3* and *-5-7* significantly correlated with gestational age [48], providing evidence that SIRTs also play a vital role during pregnancy. However, further research is needed to significantly advance the understanding of the mechanisms in which Sirtuins and NAD^+^ improve oocyte quality and early embryo development, particularly in the mare. Given what is already known about NAD^+^ and Sirtuins on oocyte quality and embryo development, propositions around the use of NAD^+^ biosynthesis to promote Sirtuin function in equine reproduction are discussed in further detail below.

## 3. NAD+, Sirtuins and Mare Fertility

### 3.1. Sirtuins and Equine Cumulus Cells

**Figure 1:**
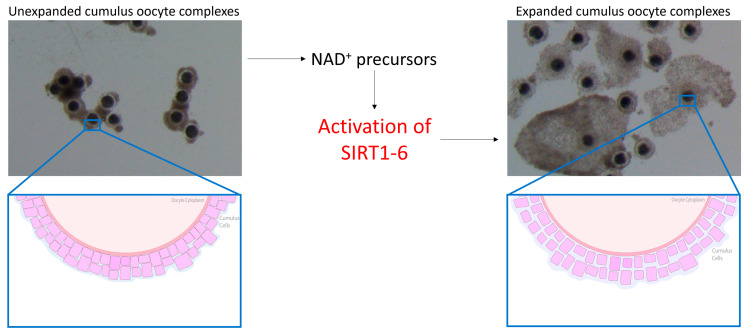
Benefits of nicotinamide adenine dinucleotide (NAD^+^) on cumulus cells in mice. The use of NAD^+^ precursors is proposed to activate SIRT1-6 in the mare, thereby promoting cumulus cell expansion and the opening of gap junctions.

The oocyte has increasingly become implicated in reduced fertility in the mare and, being seasonal breeders, this comes as no surprise. Interestingly, oocytes harvested from mare ovaries during the non-breeding season successfully reached metaphase II at the same rate as oocytes collected during the breeding season [49,50]. However, fully functional gap junctions were detected in only 21% of cumulus–oocyte complexes (COCs) [50] harvested from ovaries during the non-breeding season, indicative that communication between the oocyte and surrounding cumulus cells was adversely impacted. Gap junctions allow for the bidirectional transfer of amino acids, proteins and other factors that are important for nuclear and cytoplasmic maturation and embryonic development [51,52]. SIRT2 has previously been shown to regulate gap junctions in bovine cumulus cells through modulating the phosphorylation and deacetylation of connexin proteins [53], while SIRT1 regulated mitogen-activated protein kinase signalling pathways [54], critical for cumulus expansion [55]. Oocytes harvested from older mares have exhibited a reduction in cumulus expansion [56] in which the closing of gap junctions and therefore a decrease in oocyte–cumulus cell communication may be to blame. This suggests that inadequate cytoplasmic maturation and the inability to transfer vital amino acids and signalling factors where required may be responsible for the reduction in equine oocyte quality and developmental potential. SIRT3 and SIRT5 have been detected in human granulosa and cumulus cells [57,58], while SIRT1, 2, 4 and 6 have been detected in cumulus cells of mice [59], so it is without a doubt that SIRTs would be expressed in the cumulus cells of mares, but the detection and expression of these proteins in any reproductive tissue in horses are yet to be determined. Cetica et al. [60] previously observed an increase in enzymatic activity of nicotinamide adenine dinucleotide phosphate (NADP) in bovine cumulus cells, while NA previously enhanced granulosa cell proliferation and cumulus expansion in mice [18], indicating that increasing NAD^+^ biosynthesis through NAD^+^ precursor supplementation enhances cumulus cell function. Elevating NAD^+^ in equine cumulus cells through the use of NAD^+^ precursors may activate various SIRT proteins within both the oocyte and cumulus cells. This is proposed to promote the bidirectional communication between cumulus cells and the oocyte through the opening of gap junctions, thereby improving oocyte quality and developmental potential (Figure 1).

### 3.2. Sirtuins and Equine Mitochondrial Function

**Figure 2:**
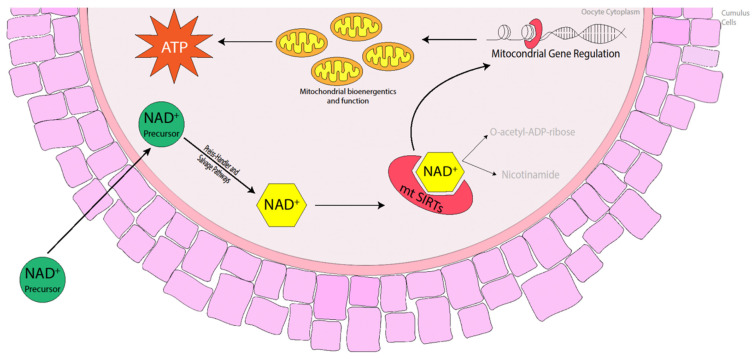
Benefits of NAD^+^ precursors in elevating NAD^+^ levels in the oocyte, activating mitochondrial Sirtuins (mtSIRTs) in mice, cattle and pigs. The activation of mtSIRTs promotes mitochondrial gene regulation, enhancing mitochondrial bioenergetics and function throughout oocyte maturation, which increases the production of ATP ready at fertilisation and during embryo development.

Commercial equine holding medium typically contains a meiotic inhibitor additive that prevents nuclear maturation but has no effect on cytoplasmic maturation, which assists in maintaining meiotic arrest. It is common practice for equine oocytes to be held overnight for logistical reasons, particularly when the source of oocytes is collected from the abattoir. Holding equine oocytes in meiosis-inhibitor-free medium prior to in vitro maturation (IVM) has not been detrimental to oocyte maturation, embryo cleavage or blastocyst formation rates. Rather, metaphase II (MII), cleavage and blastocyst formation rates were similar between oocytes placed into IVM medium immediately after collection or held overnight at 25 °C before being matured in vitro [61,62]. Oocytes isolated from older mares and matured in vitro exhibited a reduction in the number of mitochondria, many of which displayed a number of morphological abnormalities including swelling and damaged cristae [63,64], which suggests that these oocytes have a reduced capacity to produce the amount of energy necessary to promote oocyte maturation and sustain early embryo development. Fewer pregnancies were obtained when oocytes were transferred to young recipient mares from older donor mares [65], suggesting that there are oocyte inadequacies, particularly in older mares, that are often overlooked. The fact that oocytes reach MII does not necessarily mean they have full developmental potential [66]. Complete oocyte maturation in the horse may be similar to that in the pig in which cytoplasmic maturation takes longer to complete and so must begin prior to nuclear maturation before the process is halted once nuclear maturation is attained [67]. The distribution of mitochondria in the cytoplasm is one such phenomenon that takes place during cytoplasmic maturation. SIRT3, 4 and 5 are mitochondrial Sirtuin proteins [36,68] that promote ATP production and mitochondrial function in murine, porcine and bovine oocytes [45,69,70,71]. The use of nicotinamide mononucleotide (NMN) and nicotinamide riboside (NR) has previously restored ATP production and mitochondrial function in mice [10,11,19,21], cattle [14] and pigs [15], so it is highly likely that the use of NAD^+^ precursors in the mare during oocyte maturation will promote NAD^+^ biosynthesis, activating mitochondrial SIRT proteins and thereby promoting mitochondrial bioenergetics and improving mitochondrial function (Figure 2).

### 3.3. Sirtuins and Equine Nuclear Maturation/DNA Integrity

**Figure 3:**
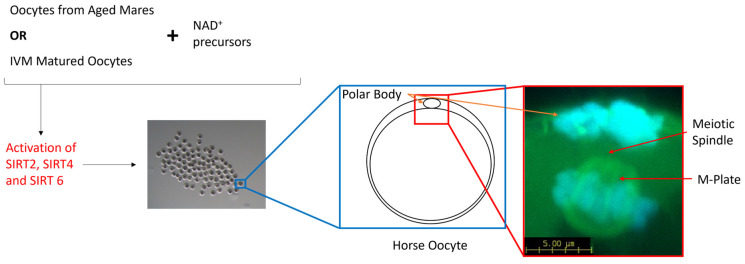
Schematic diagram representing the benefits of NAD^+^ precursors in aged mare oocytes and mare oocytes matured in vitro. NAD^+^ precursors activate SIRT-2, -4 and -6, the SIRTs responsible for the maintenance of genomic integrity and spindle formation in mammalian oocytes.

The oocyte acquires the necessary machinery to support fertilization and embryonic development when the oocyte progresses through meiosis and arrests at the second meiotic division. It is at this point where a single polar body has been extruded and the remaining DNA is highly condensed and tightly aligned along the metaphase plate. Additionally, spindles have been assembled and the individual microtubules attached to the kinetochores of the chromosomes. Up to this point, the oocyte passes through the spindle assembly checkpoint (SAC), a temporary block in the cell cycle that detects both completely unattached and improperly attached chromosomes to the spindle microtubules [72] and either signals the cell to rectify the issue or flags the cell for apoptosis. However, it appears that the SAC is much more stringent in somatic cells than in oocytes, which leaves the oocyte more prone to meiotic errors [70,71,72,73,74,75]. Furthermore, meiosis and, in particular, assembly of the meiotic spindle, is an energy-intensive process [76]. So, assembly of the spindle microtubules in combination with mitochondrial dysfunction and the resultant loss in ATP may be insufficient to promote oocyte maturation and subsequent embryo development [10,77,78]. Oocytes harvested from aged mares typically display a higher incidence of chromosome misalignment than their younger counterparts [79,80], but conflicting data persist regarding the formation of the meiotic spindle. Morphologically, the meiotic spindle and, in particular, the length of the spindle did not differ between oocytes from both younger and older mares; however, there was a greater variation in spindle length within the older cohort [80]. Similarly, oocytes matured in vitro also displayed a higher incidence of misaligned chromosomes and an increase in both the length and width of the meiotic spindle compared with in vivo-matured oocytes [81]. SIRT4 has been localised to the spindle region in mouse oocytes [82], while the inhibition of SIRT2 and SIRT6 resulted in an impaired chromosome alignment and spindle organisation in murine, bovine and porcine oocytes [45,69,83]. Defects in the meiotic spindle and the alignment of chromosomes along the metaphase plate have been ameliorated through the treatment of porcine oocytes with nicotinic acid (NA) [17] and the treatment of obese mice with intraperitoneal injections of NMN [19]. Aged mouse oocytes treated with NA, NR and NMN as a result of an elevation in NAD^+^ and a reduction in reactive oxygen species (ROS) also ameliorated spindle and chromosomal defects [10,11,14,20], potentially resulting from the subsequent activation of SIRT2, 4 or 6 (Figure 3). Therefore, the use of NAD^+^ precursors as a supplement is proposed to boost NAD^+^ levels within mare oocytes during oocyte maturation, which may ameliorate severe spindle defects and correct the alignment of chromosomes through the activation of SIRT2 and SIRT4, particularly in older mares.

### 3.4. Sirtuins and Equine IVM

**Figure 4:**
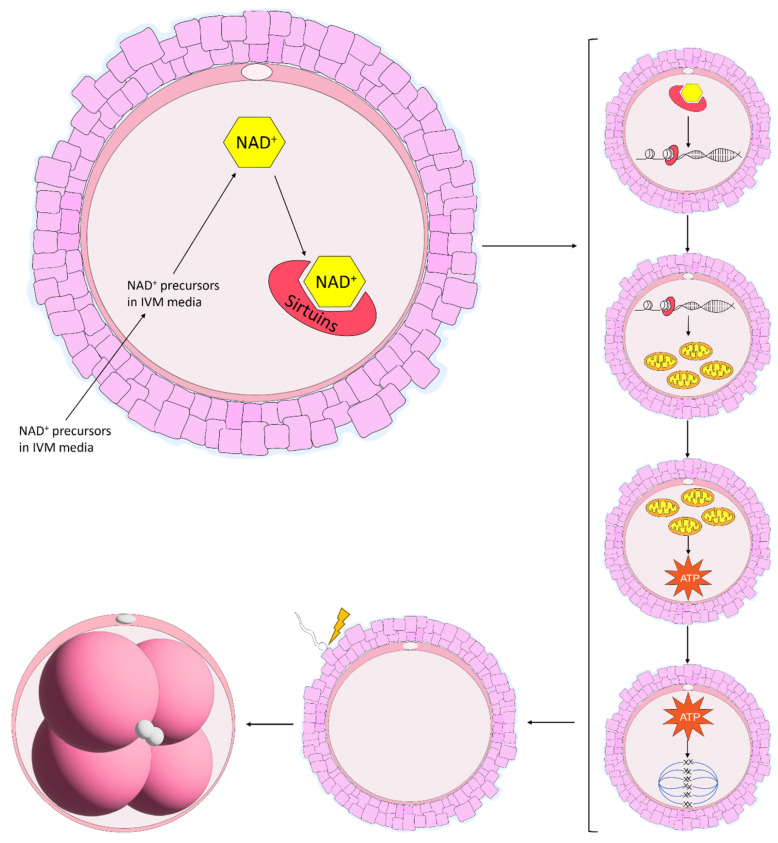
Proposed benefits of NAD^+^ precursors used in in vitro maturation (IVM) systems in the mare. In mice, NAD^+^ precursors are taken up by the oocyte and are shuttled through the various NAD^+^ biosynthetic pathways, resulting in the production of NAD^+^. NAD^+^ is then consumed by Sirtuin proteins which promote gene regulation, increasing mitochondrial function. Enhanced mitochondrial function then increases the production of ATP, improving spindle formation and chromosome alignment along the metaphase plate. Throughout this process, the oocyte gains the ability to support embryo development, an energy-expensive process following fertilisation.

The maturation of equine oocytes in vitro has been met with limited success. Several culture systems have been developed by various research groups and have been adapted from other species in which the culture media have been used successfully [84]. However, the specific requirements of equine oocytes during IVM remain unknown [85]. The scarce availability of abattoir-sourced material combined with limited follicle numbers, poor oocyte recovery rates and the time required to collect a sufficient amount of material to perform a simple experiment has prevented the upscaled development of an adequate IVM system in the horse [86]. The proportion of oocytes harvested from abattoir-sourced ovaries and matured in vitro has remained stable over the years, averaging between 50 and 60% [84,86,87]. When multiple different maturation media were compared, some returned higher embryo cleavage and blastocyst formation rates, although maturation rates did not differ, and these results were attributed to a greater efficiency in oocyte maturation [86,88]. Additionally, equine oocytes matured in vitro display lower rates of global acetylation of histone 4 at lysine residue 16 (H4K16) with a more diffuse scattering of chromosomes along the metaphase plate and an increase in the length and width of the spindle [81]. Although SIRT1 mRNA levels were not different, the expression tended to increase in oocytes matured in vitro, indicative that SIRT1 does not play a large role in histone acetylation in equine oocytes [81]. Notably, there are alterations in oocyte metabolism during IVM. Higher concentrations of glucose in the maturation media increased non-mitochondrial respiration and decreased ATP-coupled respiration [85]. Furthermore, there was a progressive loss in respiratory capacity of the oocyte over the course of IVM, suggesting that current IVM protocols are not meeting the metabolic demands of the equine oocyte [85]. Considering that the expression of proteins related to energy metabolism are differentially expressed in COCs following IVM compared with COCs matured in vivo [89], it is becoming increasingly clear that little is known regarding equine oocyte metabolism. The levels of NAD^+^ have previously been elevated in aged mouse oocytes through treatment with NAD^+^ precursors [10,11,20], which has enormous potential to improve maturation conditions in the horse. Elevating NAD^+^ through treatment with NAD^+^ precursors has improved oocyte maturation [10,11,16,20], and in particular mitochondrial function, in a range of species [45,69,70,71]. Furthermore, it is evident that Sirtuins play an extremely important role in oocyte maturation, although their role in equine oocyte maturation remains to be elucidated. Supplementing the IVM media with NAD^+^ precursors would promote the synthesis of NAD^+^, which would, in turn, activate the various SIRT proteins involved in oocyte maturation and potentially improve mitochondrial and cumulus cell function, spindle formation, chromosome alignment and oocyte metabolism, thereby improving embryo development (Figure 4).

## 4. Future Studies

It appears that the function of SIRTs is species-specific in their effects; SIRT1 has previously been correlated with an increased lifespan in *C. elegans* [90,91] and yeast [92,93] but rather plays a role in premature cellular aging during states of stress and disease [39]. Alternatively, due to its role in many biochemical and cell signalling pathways, there may be a redundancy between proteins by which one such SIRT protein will take the function of another in order to promote the required function. Similarly, there appears to be a redundancy between the NAD^+^ biosynthetic pathways, at least in yeast and bacteria where the salvage pathway will compensate for an inhibited Preiss–Handler pathway through directing the conversion of NA to nicotinamide (NAM) [94] and vice versa. Although each SIRT is said to have its own role, whether it be in ATP production, the elimination of ROS or the maintainence of genomic integrity, some SIRTs appear to exert the same or similar effects during oocyte maturation, providing support for this redundancy. Inhibitory concentrations of NAM have been shown to inhibit SIRT1-3 through a reduction in SIRT mRNA within the oocyte across a range of species; however, the effects have not completely prevented oocyte maturation from progressing [44]. As such, there is still little known regarding the requirement for NAD^+^ and Sirtuins in oocyte maturation and embryo development. The redundancy between proteins and pathways indicates that their function is vital within the cells. Given that assisted reproductive technologies and their uptake in the horse have fallen well behind those in other livestock species and in humans, the use of NAD^+^ and Sirtuins is proposed to be of great benefit, particularly with respect to in vitro technologies where poor embryo development and foaling rates follow the in vitro maturation of equine oocytes.

Future work in the mare should focus on the expression of SIRT genes and NAD^+^ levels within the various follicular compartments in vitro, including oocytes, cumulus cells, granulosa cells and follicular fluid, in order to better understand the roles and requirements of NAD^+^ and Sirtuins in mare fertility. Once these mechanisms have been clarified in the mare in vitro, studies should then focus on the application of NAD^+^ and Sirtuins in vivo to improve reproductive outcomes through non-invasive methods. Mass spectrometry has recently been used to demonstrate the metabolism of a supraphysiological dose of NA in the mare, which showed that NA was rapidly absorbed into peripheral blood within 15 min of administration [27]. Additionally, multiple blood and urine collections points indicated that NA was shuttled through the Preiss–Handler pathway with nicotinic acid adenine dinucleotide (NaAD) and NAM elevated in plasma 22 h following administration. As such, the Preiss–Handler and salvage pathways for NAD^+^ are active in mares. Further studies have also shown that NA and NMN were elevated in the follicular fluid of mares who were fed NA during the oestrous cycle [28]. Considering that older mares are more likely to be used for breeding purposes, the use of supraphysiological doses of NAD^+^ precursors is proposed to be of greater benefit in older mares with further compromised fertility, particularly when the oocyte and embryo clinical manifestations are similar to their in vitro counterparts. Other studies should also focus on the potential effects of supplementing other, potentially more potent NAD^+^ precursors on the elevation of NAD^+^ in the various follicular compartments, and then analysing the expression of SIRTs following supplementation before conducting a fertility trial to determine whether dietary supplements can assist with improving oocyte quality and preventing early embryonic death in the mare. Finally, these NAD^+^ precursors should also be trialled during oocyte IVM in the horse to determine whether these supplements can increase NAD^+^ production and SIRT activation in oocytes for ultimate use in improving IVM and in vitro fertilisation protocols in the horse.

## 5. Conclusions

This review discussed the production of NAD^+^ within the cell, its role in activating SIRTs and the described effects on fertility. Issues related to mare fertility and propositions about how NAD^+^ and Sirtuins may be involved are an area of great interest, and the scope for improvements to reproductive fecundity of the species shows enormous potential. Lastly, the future work that should take place in order to further our understanding of NAD^+^, Sirtuins and their impact on fertility in the mare was discussed. Mares suffer from a reduction in reproductive performance in comparison with their livestock counterparts, and due to the ban on assisted reproductive technologies in the Thoroughbred industries around the world combined with a lack of uptake of these technologies, fertility in the horse has suffered drastically. The roles of NAD^+^ and Sirtuins in the horse have yet to be investigated, but offer great potential at improving oocyte quality, especially during IVM programs, and may, in time, translate to a reduction in early embryonic death. Studies in mice, cattle and pigs have shown that NAD^+^ biosynthesis enhances oocyte quality and embryo development, so it is proposed that mare fertility would greatly benefit.

## Data Availability

Data sharing not applicable.
